# DNA methylation pattern changes upon long-term culture and aging of human mesenchymal stromal cells

**DOI:** 10.1111/j.1474-9726.2009.00535.x

**Published:** 2010-02

**Authors:** Simone Bork, Stefan Pfister, Hendrik Witt, Patrick Horn, Bernhard Korn, Anthony D Ho, Wolfgang Wagner

**Affiliations:** 1 Department of Medicine V, University of Heidelberg69120 Heidelberg, Germany; 2 Heidelberg Academy of Sciences and Humanities69117 Heidelberg, Germany; 3 Department of Pediatric Oncology, Hematology and Immunology, University of Heidelberg69120 Heidelberg, Germany; 4 Molecular Genetics of Pediatric Brain Tumors, German Cancer Research Center (DKFZ)69120 Heidelberg, Germany; 5 Genomics and Proteomics Core Facility, German Cancer Research Center (DKFZ)69120 Heidelberg, Germany; 6 Helmholtz-Institute for Biomedical Engineering, Aachen University Medical School52074 Aachen, Germany

**Keywords:** aging, DNA methylation, human mesenchymal stromal cells, long-term culture, pyrosequencing, senescence

## Abstract

Within 2–3 months of *in vitro* culture-expansion, mesenchymal stromal cells (MSC) undergo replicative senescence characterized by cell enlargement, loss of differentiation potential and ultimate growth arrest. In this study, we have analyzed DNA methylation changes upon long-term culture of MSC by using the HumanMethylation27 BeadChip microarray assessing 27 578 unique CpG sites. Furthermore, we have compared MSC from young and elderly donors. Overall, methylation patterns were maintained throughout both long-term culture and aging but highly significant differences were observed at specific CpG sites. Many of these differences were observed in homeobox genes and genes involved in cell differentiation. Methylation changes were verified by pyrosequencing after bisulfite conversion and compared to gene expression data. Notably, methylation changes in MSC were overlapping in long-term culture and aging *in vivo*. This supports the notion that replicative senescence and aging represent developmental processes that are regulated by specific epigenetic modifications.

## Introduction

Mesenchymal stromal cells (MSC) are precursors for mesodermal cell lineages such as osteocytes, chondrocytes, and adipocytes. They can be isolated from various tissues by plastic adherent growth under specific culture conditions. MSC are heterogeneous, and reliable markers for the definition of the multipotent subset of ‘mesenchymal stem cells’ remain to be elucidated (Horwitz *et al.*, 2005; Dominici *et al.*, 2006; Wagner & Ho, 2007). Nevertheless, the ease of isolation and the possibility of culture expansion to very high cell numbers as well as the absence of critical side effects of MSC in clinical trials raise hopes for a variety of therapeutic applications (Wagner & Ho, 2007).

Mesenchymal stromal cells have a restricted lifespan *in vitro* such as any other somatic cell and can therefore only be expanded for a limited number of cell divisions before entering a senescent state and unequivocally stopping proliferation. This is accompanied by enlargement and morphologic changes referred to as ‘fried egg morphology’. Furthermore, many groups have shown that long-term culture of MSC impairs their differentiation potential (Banfi *et al.*, 2000; Baxter *et al.*, 2004; Bonab *et al.*, 2006; Noer *et al.*, 2007; Wagner *et al.*, 2008). Therefore, the senescent state needs to be taken into account for quality control of MSC in cellular therapy.

The phenomenon of replicative senescence was first described in the 1960s by Leonard Hayflick (Hayflick, 1965) and since then a scientific discussion is ongoing whether the so-called ‘Hayflick limit’ resembles the aging process of the whole organism. We have recently demonstrated that replicative senescence induces reproducible gene expression changes in MSC (Wagner *et al.*, 2008; Schallmoser *et al.*, 2009). Interestingly, these changes closely resemble age-associated gene expression changes in adult stem and progenitor cells of young vs. elderly donors (Wagner *et al.*, 2009). This overlap in differential gene expression indicates that long-term culture and aging might be regulated by similar mechanisms.

Two different basic principles are conceivable for cellular aging: (i) it might either be due to accumulation of damage that leads to cellular deterioration (Kirkwood & Austad, 2000) or (ii) it results from a cellular program that is regulated by a biological clock (de Magalhaes & Church, 2005; Prinzinger, 2005). Various molecular pathways have been shown to be relevant for the process of aging and senescence such as telomere shortening, DNA damage, accumulation of the cyclin-dependent kinase inhibitor p16INK4a and oxidative stress (Kiyono *et al.*, 1998; O’Hare *et al.*, 2001; Di Donna *et al.*, 2003; Ho *et al.*, 2005; Janzen *et al.*, 2006). Furthermore, it has been proposed that epigenetic changes such as DNA methylation play a central role in senescence and aging (Wilson & Jones, 1983; Young & Smith, 2001; Nilsson *et al.*, 2005; Chambers *et al.*, 2007; Shibata *et al.*, 2007; Suzuki *et al.*, 2008).

DNA methylation is the best characterized epigenetic modification. CpG dinucleotides in the mammalian genomic DNA may be methylated at cytosine moieties. Upon replication the same methylation pattern is established on the newly synthesized DNA strand by DNA methyltransferase 1 (DNMT1). Thereby, epigenetic modifications are inherited to both daughter cells (Jaenisch & Bird, 2003). Methylation may impact gene transcription by direct interference with transcription factors or with methyl-CpG-binding proteins that modify histones and thereby inactivate the respective promoter region. Other authors have previously suggested that methylation levels gradually decrease upon long-term culture (Wilson & Jones, 1983; Nilsson *et al.*, 2005). This study is based on the hypothesis that long-term culture and aging are associated with epigenetic modifications at specific CpG sites.

## Results

### Long-term culture of MSC

Mesenchymal stromal cells were isolated from bone marrow aspirates from young donors (21–50 years) or from bone marrow aspirates from elderly donors (53–85 years) upon hip fracture as previously described (Wagner *et al.*, 2009). All cell preparations fulfilled the criteria of MSC such as typical growth morphology, immunophenotype and *in vitro* differentiation potential (Fig. S1) (Dominici *et al.*, 2006; Wagner & Ho, 2007). Within 2–3 months of long-term culture the cells underwent replicative senescence accompanied by a decreased adipogenic differentiation potential and an increased propensity for osteogenic differentiation (Wagner *et al.*, 2008) whereas no spontaneous differentiation was observed under nondifferentiation conditions even at later passages. The proliferation rate decreased gradually until the cells finally stopped to proliferate (Fig. S2A). These senecent passages were associated with expression of senescence-associated beta-galactosidase (SA-beta-gal) and increased p16 gene expression (Fig. S3).

### Replicative senescence affects DNA methylation patterns

**Fig. 1:**
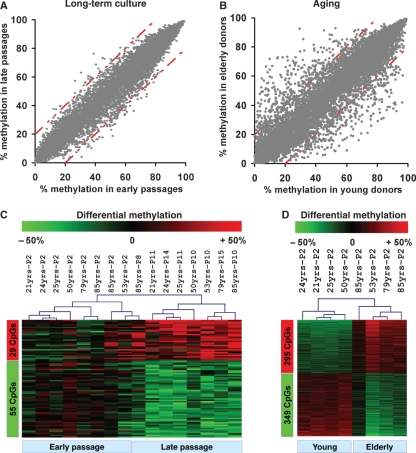
CpG methylation changes upon long-term culture and aging. DNA methylation at 27 578 different CpG sites was analyzed using the HumanMethylation27 BeadChip microarray. Scatter plots represent the mean methylation levels of mesenchymal stromal cells from early passage vs. late passage (A) or of young vs. elderly donors (B). Differential methylation of more than 20% was considered to be relevant and this is demonstrated by the dashed red lines. Heat map presentation of differentially methylated CpGs in long-term culture (C) and aging (D) are shown. Notably, unsupervised hierarchical clustering of CpG sites that revealed differential methylation in long-term culture revealed an age-associated relationship of methylation profiles.

To determine effects of long-term culture on DNA methylation patterns we harvested MSC of early passages (2^nd^ passage; P2) and late passages (P8–P15) from eight donors. Methylation status at 27 578 CpG sites was simultaneously analyzed using the HumanMethylation27 BeadChip microarray (Illumina). Scatter plot analysis of methylation in early vs. late passages revealed that DNA methylation remains overall constant upon MSC culture expansion (Fig. 1A). However, several CpGs showed a highly significant degree of differential methylation between early and late passage in all eight MSC samples. Only differential methylation of more than 20% was considered to be relevant and these CpGs were considered for further analysis. Twenty-nine CpGs were hypermethylated upon long-term culture whereas 55 CpGs were hypomethylated (Fig. 1C; Table S1). Hypermethylated CpGs were distributed in the promoter regions and within 23 nonredundant and functionally characterized genes including distal-less homeobox 5 (*DLX5*), runt-related transcription factor 3 (*RUNX3*), and chromosome 10 open reading frame 27 (*C10orf27*). Hypomethylated CpGs were localized in the promoter regions and within 43 protein-coding genes including cycline-dependent kinase inbibitor 2B (*CDKN2B*), bone morphogenic proteins 10 and 15 (*BMP10/15*), left-right determination (*LEFTY1*), and distinct CpG sites within the *RUNX3* promoter. The function of all differentially methylated genes was further classified by Gene Ontology analysis. Hypermethylated genes were significantly over-represented in the functional categories cell-matrix adhesion, embryonic morphogenesis and development whereas hypomethylated genes were rather associated with epidermal differentiation (Table S2). There was only a moderate enrichment of methylation changes in specific chromosome regions: Three of the hypermethylated genes were located at chromosome band 10q22 (*P* = 0.0003) whereas four of the hypomethylated genes were enriched at chr21q22 (*P* = 0.0012). Overall, our results inevitably demonstrate that long-term culture induces reproducible and significant methylation changes at specific CpG sites and especially seem to affect genes involved in development.

### DNA methylation changes upon aging

To further elucidate methylation changes in MSC upon aging, we next analyzed methylation profiles in MSC that were taken from the second passage of young or elderly donors, respectively. Several CpG sites revealed age-associated methylation changes (Fig. 1B). Two hundred and ninety-five CpGs were hypermethylated, whereas 349 CpGs were hypomethylated upon aging (Fig. 1D; Table S3). Genes associated with CpGs that were hypermethylated upon aging included *HOXA2*, *HOXA5*, *HOXA6*, *HOXB2*, *DLX5*, aristaless-like homeobox 4 (*ALX4*), T-box 5 isoform 2 (*TBX5*), *RUNX2*, *RUNX3*, O-6-methylguanin-DNA methyltransferase (*MGMT*) and *C10orf27*. Genes associated with CpGs that were hypomethylated upon aging included *HOXB1*, *HOXB3*, *HOXB4*, *HOXB6*, *HOXD3*, *HOXD12*, paired-like homeodomain transcription factors *PITX1* and *PITX2*, sirtuin 6, *BMP4*, mesoderm specific transcript (*MEST*), paired box genes *PAX1*, *PAX8*, *PAX9*, *TBX1*, *S100A4*, and also *RUNX3* and *ALX4*. Differentially methylated genes were further classified by Gene Ontology analysis. Hypermethylated genes were significantly over-represented in various signal transduction pathways and limb morphogenesis. In contrast to this, hypomethylated genes were most significantly over-represented in genes involved in sequence-specific DNA binding and developmental processes (Table S4). Furthermore, there was an enrichment of hypermethylated genes at the chromosome regions chr21q22 (9 genes; *P* = 0.0006), chr2p25 (5 genes; *P* = 0.0011), chr6q27 (4 genes; *P* = 0.0013), and chr12q24 (7 genes; *P* = 0.0073). Hypomethylated genes upon aging were enriched at chr20q13 (9 genes; *P* = 0.0032). Interestingly, methylation changes were highly enriched in genes with simian-virus-40-protein 1 (SP1) binding sites (hypermethylation: 15 of 258 genes, *P* = 7.8 × 10^−11^ and hypomethylation: 31 of 273 genes, *P* = 6.0 × 10^−5^).

### Validation of differentially methylated genes by pyrosequencing

**Fig. 2:**
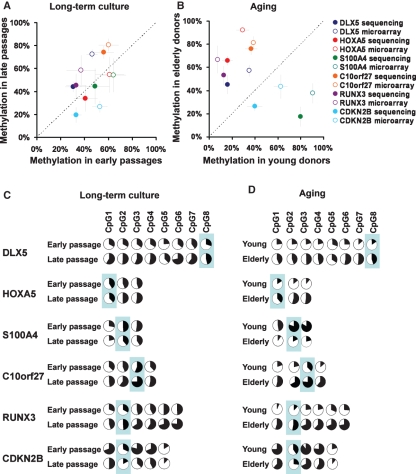
Pyrosequencing of differentially methylated CpGs. For six CpG sites differential methylation of the HumanMethylation27 BeadChip (open circles) was validated by pyrosequencing (filled circles) (all 16 samples, ± SEM). Overall, methylation levels were lower in pyrosequencing but there was a striking correlation in differential methylation between the two methods for long-term culture (A) and aging (B). This differential methylation was also observed in CpGs in close vicinity to the analyzed CpG site represented on the microarray (blue box; C, D). The percentage of methylation at each CpG site is indicated in black.

Differential methylation of six selected CpG sites (*DLX5*, *HOXA5*, *S100A4*, *C10orf27*, *RUNX3*, and *CDKN2B*) was validated by pyrosequencing after bisulfite conversion. Candidate genes were selected based on the correlation between gene expression and CpG island methylation as well as their attributed biological function. Methylation levels were overall slightly lower in pyrosequencing results as compared with microarray analysis. However, differential methylation upon long-term culture or upon aging was very consistent between the two methods (Fig. 2A,B). Moreover, differential methylation was also verified in three additional independent donor samples (Fig. S4A). Comparative analysis of different passages revealed that methylation changes are continuously acquired with every passage upon long-term culture (Fig. S4B). Notably, also CpGs in close neighborhood to the CpG represented on the microarray revealed very similar methylation patterns (Fig. 2C,D; Fig. S4C,D). This demonstrates that methylation changes are not restricted to individual CpG sites but rather affect whole CpG islands.

### Association of methylation changes in long-term culture and aging

**Fig. 3:**
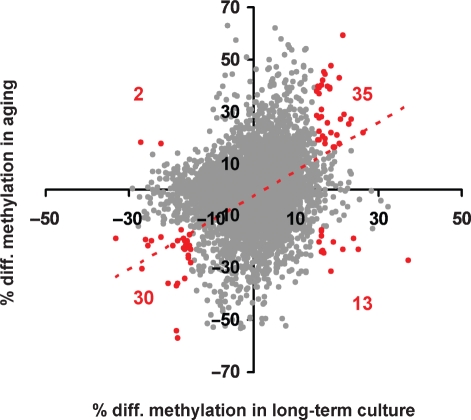
Comparison of differential methylation upon long-term culture and aging. Methylation changes upon long-term culture were plotted against methylation changes upon aging. CpGs with a more than 15% differential methylation in both comparisons are depicted (red spots). These differentially methylated CpGs revealed that DNA methylation changes upon long-term culture and aging are overlapping (correlation coefficient R = 0.61; *P* = 4.6 × 10^9^).

We have previously shown that long-term culture and aging have similar effects on the gene expression profile of MSC (Wagner *et al.*, 2008, 2009). Therefore, we hypothesized that these two processes might also reveal an association in DNA methylation changes. Only 13 CpGs were more than 20% differentially methylated in both comparisons and therefore we have chosen a less stringent cutoff of 15% differential methylation resulting in 80 CpGs (Fig. 3). These demonstrated a correlation in long-term culture and aging (*R* = 0.61) and chi-square analysis revealed a statistical significance of 4.6 × 10^−9^. Hence, it might be speculated that senescence-associated changes are more pronounced in samples from younger donors. Indeed, the number of more than 20% differentially methylated CpG sites upon long-term culture was 24% higher in younger vs. elderly donors (248 CpG sites vs. 189 CpG sites). These data indicate that long-term culture and aging resemble two processes with related epigenetic modifications.

### Association of DNA methylation and gene expression

**Fig. 4:**
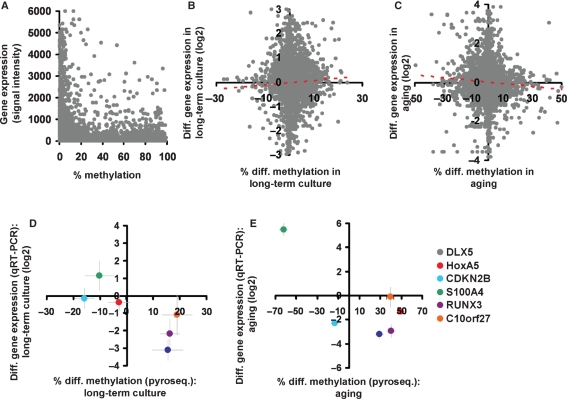
Correlation of DNA methylation and gene expression. DNA methylation data of the HumanMethylation27 BeadChip was matched with Affymetrix mRNA expression profiles. As expected, signal intensity in gene expression data revealed higher expression of nonmethylated genes (A). However, differential methylation upon long-term culture (B) or aging (C) was not necessarily reflected in differential gene expression. Six differentially methylated genes that have been analyzed by pyrosequencing were further analysed by quantitative RT-PCR and in this subset hypermethylation coincides with lower gene expression and *vice versa* (D, E) (all 16 samples, ± SEM).

DNA methylation profiles were integrated with gene expression data to determine the impact of epigenetic modifications on mRNA expression. For each CpG on the HumanMethylation27 BeadChip the corresponding gene was matched to the Affymetrix mRNA gene expression data by gene symbols (Wagner *et al.*, 2009). Signal intensity in gene expression data was used as an indicator for gene expression levels. As expected, nonmethylated genes revealed higher gene expression levels than methylated genes (Fig. 4A). Subsequently, we tested if differential methylation was also reflected in differential gene expression. Overall the two microarray platforms did not reveal a clear correlation between differential CpG methylation and differential mRNA expression in long-term culture or aging (Fig. 4B,C). This might be due to the different sensitivity of the two techniques: 20% differential methylation can be detected, whereas 20% differential gene expression can not be reliably discerned. We have selectively addressed gene expression changes in genes that have been analyzed by pyrosequencing and that revealed higher methylation changes. mRNA expression of these genes (*DLX5*, *HOXA5*, *CDKN2B*, *S100A4*, *RUNX3*, and *C10orf27*) was analyzed by quantitative RT-PCR. For these selected genes hypermethylation coincided with lower gene expression and *vice versa* (Fig. 4D,E).

## Discussion

Epigenetic modifications play a central role in differentiation and development. This study indicates that modification of DNA methylation is also involved in replicative senescence upon long-term culture of MSC. Furthermore, a similar pattern of differential methylation was observed between MSC from young and elderly donors. These results support the perception that replicative senescence and aging are not only due to stochastic accumulation of cellular defects but rather represent a developmental program. On the other hand, the process is overlaid by stress-induced senescence changes e.g. due to high oxygen content or the artificial environment. Furthermore, the cellular aging of MSC might be accompanied by a different composition of heterogeneous subpopulations and loss of self-renewal potential under *in vitro* conditions. Hence, senescence of MSC has many facets that need to be further elucidated (Wagner *et al.*, review submitted).

Methylation was analyzed by using the novel HumanMethylation27 BeadChip representing 27 578 CpGs that are associated with promoter regions of more than 13 500 annotated genes. This array was designed to cover CpG dinucleotides in the promoter region of genes and therefore is clearly biased toward the assessment of gene-specific methylation. Differential CpG methylation was highly reproducible in different donor samples and was verified by pyrosequencing for all sequences tested. Thus, this technique facilitates reliable profiling of global methylation patterns.

Other authors have reported that global DNA methylation levels decrease upon long-term culture (Wilson & Jones, 1983; Young & Smith, 2001). Therefore, it has been speculated that maintenance of DNA methylation by DNA methyltransferase 1 (DNMT1) is incomplete resulting in a gradual decrease of DNA methylation with repeated cell cycles (Nilsson *et al.*, 2005). However, our results did not reveal an overall decrease in DNA methylation levels. In fact, methylation remained constant over the whole culture period at most CpG sites represented on the BeadChip. To our knowledge this study demonstrates for the first time that only specific CpG islands become differentially methylated upon long-term culture. DNA methylation changes upon aging may also depend on individual differences although differential methylation was verified by pyrosequencing in independent donor samples. It is also likely, that age-related changes are influenced by the different cellular composition upon aging as well as by the different tissue and isolation technique.

Methylation changes in our study were often associated with homeobox genes. Expression of these genes is tightly regulated under temporal control during vertebrate development. It has been shown that chromatin modifications play an important role for regulation of the ‘Hox clock’ that coordinates body patterning (Illingworth *et al.*, 2008; Soshnikova & Duboule, 2009). Furthermore, age-associated gene expression changes have been described in homeobox genes (Stelnicki *et al.*, 1998; Wagner *et al.*, 2009) and it is conceivable that they are involved in the timing of aging. Exemplarily, we have verified differential methylation for *HOXA5* and *DLX5* upon long-term culture and aging by pyrosequencing. The homeobox gene *DLX5* is also involved in differentiation toward the osteoblastic lineage (Lee *et al.*, 2005). Therefore, hypermethylation and decreased gene expression of *DLX5* might be relevant for the impaired differentiation capacity of MSC upon long-term culture. It has been shown that methylation changes at specific CpG sites can affect differentiation of MSC (Noer *et al.*, 2007; Zimmermann *et al.*, 2008). In addition, age-related methylation changes have been described. Several tumor suppressor genes are hypermethylated upon aging with potential implications for cancer susceptibility in the elderly (So *et al.*, 2006). Among these was *RUNX3* that revealed age-associated changes in our analysis. Another differentially methylated gene was the cycline-dependent kinase inbibitor 2B (*CDKN2B*) that has been implicated in senescence in our previous work (Schallmoser *et al.*, 2009).

Several genes such as *RUNX3* and *ALX4* were more than 20% hypermethylated at specific CpG sites and more than 20% hypomethylated in others. These antagonistic modifications within the same promoter region indicate that DNA modifications are restricted to relatively short and specific sequences. On the other hand, pyrosequencing revealed that methylation changes are not restricted to unique CpG sites but also include neighboring cytidines of the same CpG island. It has been suggested that senescence can also be induced in immortal cells by treatment with 5aza-2deoxycytidine (5AZA) (Vogt *et al.*, 1998). However, it is difficult to discern toxic effects of 5AZA. The complex pattern of hypermethylation and hypomethylation observed in this study might explain why replicative senescence cannot be controlled by demethylating agents.

*De novo* methylation is facilitated by DNA methyltransferases DNMT3A and DNMT3B but it is mostly unknown how the extent of *de novo* methylation is regulated and where it takes place. Active DNA hypomethylation is also considered to occur but a specific enzyme that removes the methyl group from 5-methylcytosine has not yet been described. It has been suggested that DNA methyltransferases exhibit dual actions in CpG methylation and demethylation of 5mCpGs through deamination (Kangaspeska *et al.*, 2008; Metivier *et al.*, 2008). DNA methylation patterns may vary drastically within CpG islands and only methylation of a defined core-region silences gene expression (Gonzalgo *et al.*, 1998; Deng *et al.*, 1999; Ushijima & Okochi-Takada, 2005). We have shown that age-associated hypermethylated and hypomethylated genes are enriched in SP1 transcription factor binding sites and it has been proposed that binding of the SP1 transcription factor protects CpG islands from methylation (Brandeis *et al.*, 1994; Macleod *et al.*, 1994). ‘Seeds of methylation’ is another interesting mechanism that leads to increasing methylation of CpG islands. This hypothesis is based on the observation that little methylation is induced in an initially nonmethylated region whereas a high degree of methylation is induced in a sparsely methylated region (Song *et al.*, 2002; Stirzaker *et al.*, 2004). This mechanism might serve as a cell-cycle counter.

It is commonly accepted that DNA methylation silences gene expression. This is also reflected by the correlation of the two different levels investigated in our study: nonmethylated genes revealed higher signal intensity in mRNA expression profiling. However, differential DNA methylation at specific CpG sites does not necessarily correlate with observed gene expression changes. Relevant fold-changes in gene expression can only be expected if the CpG sites are predominantly methylated. It is also conceivable that selective modification of specific promoter regions either activates or inactivates binding of transcription regulators.

We have previously described age-related gene expression changes in human stem and progenitor cells and observed a moderate but significant concordance in the expression profiles upon aging *in vivo* and replicative senescence *in vitro* (Wagner *et al.*, 2009). However, the proliferation rate was not higher in MSC from younger donors and they did not reach a higher number of cumulative population doublings. This might be attributed to the relatively low number of samples per group as other authors reported an inverse relationship between donor age and the replicative life span *in vitro* for fibroblasts or MSC (Schneider & Mitsui, 1976; Stenderup *et al.*, 2003; Mareschi *et al.*, 2006; Shibata *et al.*, 2007). Also the doubling time of either freshly isolated marrow cells or MSC of early passage has been described to be significantly lower in samples from elderly people (Zhou *et al.*, 2008) whereas this was not observed in our study. Analysis of age-specific changes might be affected by the different sources for MSC that were available for the two age groups. MSC were either isolated from human bone marrow from the iliac crest or from the femoral head. There were no differences in growth, cell morphology, immunophenotype or differentiation potential (Wagner *et al.*, 2009). Thus, MSC from BM and HIP fulfilled the criteria that are commonly used for definition of MSC although these cell preparations are heterogeneous and may comprise different subpopulations (Dominici *et al.*, 2006; Wagner & Ho, 2007). However, we can not exclude that some of the methylation changes are due to the different bone marrow regions. Notably, however, there was a relevant overlap of methylation changes upon long-term culture and aging of MSC. Furthermore, there is a significant concordance of our age-related changes in MSC and age-associated methylation changes in ovarian cancer samples (Teschendorff *et al.*, manuscript in preparation). Further integrative analysis of methylation profiles of different cell types and tissues will help to discern the existence of a distinct methylation signature for aging.

Bone marrow is a complex tissue with various different cell types. Hence, it is apparent that aging and replicative senescence are not identical despite molecular similarities of the two processes. Long-term culture has been shown to impair differentiation potential of MSC, especially of adipogenic differentiation (Baxter *et al.*, 2004; Bonab *et al.*, 2006; Wagner *et al.*, 2008). By contrast, other authors demonstrated that aging activates adipogenic differentiation and suppresses osteogenic differentiation (Moerman *et al.*, 2004; Zhou *et al.*, 2008). These authors also demonstrated that expression of the master transcription factor for osteoblast differentiation RUNX2 and DLX5 decreased upon aging and this is in line with higher methylation as observed in our data. It is conceivable, that the higher propensity for adipogenesis in BM of elderly people results in a lower number of osteogenic progenitor cells.

Aging is not an inevitable fate of all cells. Our germ line cells and their progeny are sustained through the generations and embryonic stem cells as well as induced pluripotent stem cells do not show signs of replicative senescence upon long-term culture (Zeng, 2007). It is therefore conceivable, that the loss of pluripotency is directly accompanied with limitation of the replicative live span (Marciniak-Czochra *et al.*, 2009). Both mechanisms might be regulated by similar epigenetic modifications that govern differentiation and development. Our results demonstrate that specific CpG sites become either methylated or demethylated upon long-term culture. Analysis of these specific CpG sites might facilitate monitoring of the replicative lifespan of cells. It will be of central interest to further increase our understanding of the mechanisms regulating senescence-associated and age-associated epigenetic modifications.

## Experimental procedures

### Isolation of MSC from human bone marrow

Mesenchymal stromal cells were isolated from human bone marrow that was either taken from aspirates from healthy donors for allogeneic transplantation (BM) or from the caput femoris from elderly patients undergoing femoral head prosthesis (HIP) as described in our previous work (Wagner *et al.*, 2009). All samples were taken after written consent using guidelines approved by the Ethic Committee on the Use of Human Subjects at the University of Heidelberg. The standardized culture conditions have been described in detail before (Reyes *et al.*, 2001; Wagner *et al.*, 2005).

### Expansion and sampling of MSC

Mesenchymal stromal cells were cultured for 2–3 month by serial passaging until the cells reached a senescent state and stopped proliferation. Culture conditions and sampling have been described in our previous work (Wagner *et al.*, 2008, 2009). In brief, 7–10 days after the initial seeding of MNC the colonies were trypsinized and re-plated in a new culture flask (passage 1, P1). Upon sub-confluent growth (70%), cells were re-plated at a density of 10^4^cells cm^−2^. Cells were counted at every passage by a Neubauer counting chamber and the cumulative population doublings were calculated (Cristofalo *et al.*, 1998). As cell numbers were first determined at P1, the cumulative doubling number was first calculated for P2. From P2 onward, there were enough cells for simultaneous expansion of one fraction and harvesting another fraction for subsequent analyses: 10^6^ cells were lysed in TRIzol for RNA isolation, 10^6^ cells were cryopreserved for immunophenotyping and *in vitro* differentiation assays (Wagner *et al.*, 2008, 2009) and 10^6^ cells were pelleted and stored by −80 °C for subsequent DNA isolation.

### Immunophenotypic analysis of MSC and *in vitro* differentiation of MSC

Immunophenotypic characterization of MSC preparations was performed on a three-color FACScan (Becton Dickinson [BD], San Jose, CA, USA) with a five-color upgrade (Cytek Dev. Inc., Fremont, CA, USA) using the commonly used panel of antibodies as described before (Wagner *et al.*, 2009): CD13-allophycocyanin (APC, clone WM15, BD), CD29-fluorescein isothiocyanate (FITC, MEM-101a, abcam, Cambridge, UK), CD31-FITC (BD), CD34-phycoerythrin (PE, 8G12, BD), CD44-PE (g44-26, BD), CD45-FITC (2D1, BD), CD73-PE (AD2, BD), CD90-PE (G7, BD), CD105-PE (MHCD10504, BD), CD146-PE (P1H12, BD), CD166-PE (3A6, BD), CD184-PE (12G5, BD). Osteogenic and adipogenic differentiation of MSC was induced as previously described (Wagner *et al.*, 2009). After 21 days osteogenic differentiation was analyzed by Alizarin red staining and adipogenic differentiation by Oil Red-O staining**.**

### Senescence-associated beta-galactosidase staining

Expression of pH-dependent senescence-associated beta-galactosidase (SA-beta-gal) activity was analyzed simultaneously in different passages of MSC of young and elderly donors using the SA-beta-gal staining kit (Cell Signaling Technology, Boston, MA, USA).

### DNA isolation and bisulfite conversion

Genomic DNA of 10^6^ MSC was isolated using the QIAGEN DNA Blood Midi-Kit. DNA quality was assessed with a NanoDrop ND-1000 spectrometer (NanoDrop Technologies, Wilmigton, DE, USA) and average fragment length was assessed by gel electrophoresis. Genomic DNA (500 ng) from each sample was bisulfite converted using the EZ-96 DNA Methylation Kit (Zymo research Corporation, Orange, CA, USA). This step leads to the deamination of nonmethylated cytosines to uracils, while methylated cytosines are refractory to the effects of bisulfite and remain cytosine. After bisulfate conversion each sample was whole genome amplified and enzymatically fragmented.

### Profiling of CpG methylation

The HumanMethylation27 BeadChip (Illumina, San Diego, CA, USA) uses Infinium technology, previously described for SNP genotyping (Steemers *et al.*, 2006), to perform genome-wide screening of DNA methylation patterns. In detail, the HumanMethylation27 panel targets 27 578 unique CpG sites located within the proximal promoter regions of transcription start sites of 14 475 consensus coding sequencing (CCDS) in the NCBI Database (Genome Build 36). In addition, 254 assays cover 110 miRNA promoters. On average, two assays were selected per CCDS gene and from 3 to 20 CpG sites for >200 cancer-related and imprinted genes. Further specifications of the platform can be found at http://www.illumina.com/products/infinium_humanmethylation27_beadchip_kits.ilmn. About 200 ng DNA was applied per BeadChip according to the manufacturer’s instructions. During hybridization, the DNA molecules anneal to two different bead types with locus-specific DNA oligomers – one corresponds to the methylated (C) and the other to the unmethylated (T) state. Allele-specific primer annealing is followed by single-base extension using DNP- and Biotin-labeled ddNTPs. After extension, the array is fluorescently stained, scanned, and the intensities of the unmethylated and methylated bead types measured. Initial data analysis was performed with the BeadStudio Methylation Module. DNA methylation values, described as beta values, are recorded for each locus in each sample. Only few CpG sites did not reveal reliable signals, as determined by their detection *P*-value, and were therefore excluded from further analysis. Raw data were quantile normalized. Statistical analysis and clustering by Euclidian distance was performed using the MultiExperiment Viewer (MeV, TM4) (Saeed *et al.*, 2003). For further analysis we have selected CpG sites with more than 20% differential methylation. These were further analyzed to calculate mean methylation, differential methylation and student’s *t*-test. Furthermore, genes associated to the differentially methylated CpG sites were categorized by Gene Ontology analysis using GoMiner software (http://discover.nci.nih.gov/gominer/). Chromosomal distribution and transcription factor binding was assessed by Gene Set Enrichment Analysis (Subramanian *et al.*, 2005). The complete CpG methylation values have been deposited in NCBIs Gene Expression Omnibus (GEO, http://www.ncbi.nlm.nih.gov/geo/) and are accessible through GEO Series accession number GSE17448.

### Pyrosequencing

Methylation changes of six selected CpG sites were validated by pyrosequencing. Independent bisulfite treatment and pyrosequencing assays were performed by Varionostic GmbH (Ulm, Germany). In brief, bisulfite converted DNA was subjected to PCR amplification. Primers are provided in Table S5. 40–45 μL of PCR product was immobilized to 3 μL Streptavidin SepharoseTM HP beads (GE Healthcare, Piscataway, NJ, USA) followed by annealing to 2 μL sequencing primer (5 μM) for 2′ at 80 °C. CpG analysis was done with Pyro Q-CpG software, and differential methylation was calculated as described above.

### Gene expression profiling

In this study we have combined DNA methylation data with gene expression profiles of our previous work (Wagner *et al.*, 2008, 2009). Gene expression profiles have been analyzed by the U133_Plus_2.0 Affymetrix chip and two different data sets were used for this comparison.

1. *MSC-replicative senescence* (Wagner *et al.*, 2008): MSC of three different donors were analyzed at passage 2 (P2) and at the senescent passage (Px). This resulted in a set of six hybridizations. Raw data were quantile normalized and log_2_ ratios were calculated vs. P2 of the corresponding donor sample. The complete microarray data are accessible through GEO Series accession number GSE9593.
2. *MSC-donor age* (Wagner *et al.*, 2009): MSC of four younger donors (21–50 years) and four elderly donors (53-85 years) were harvested at the second passage. These are the same donor samples that have also been used for methylation profiling. Raw data were quantile normalized and log_2_ ratios were calculated between the different age groups. The complete microarray data are accessible through GEO Series accession number GSE12274.

Gene expression data was matched to HumanMethylation27 BeadChip by gene symbols (HUGO names). For comparative analysis we have only considered probe sets that were detected as present in more than 50% of the hybridizations in gene expression analysis.

### Quantitative real-time PCR analysis

Quantification of mRNA expression for candidate genes was performed by real-time quantitative PCR (QRT-PCR) using the ABI PRISM® 7700HT Sequence Detection System Instrument (Applied Biosystems, Applera Deutschland GmbH, Darmstadt, Germany). Total RNA was isolated and reverse transcribed as described in our previous work (Wagner *et al.*, 2009). Primers were obtained from Biospring (Frankfurt, Germany) (Table S5). QRT-PCR reactions were performed with the power SYBR® green PCR master mix in a MicroAmp optical 96-well reaction plate with a ABI PRISM® 7700HT sequence detector (Applied Biosystems) according to the manufacturer’s instructions. Gene expression levels were normalized to GAPDH expression, which was used as a housekeeping gene.

## Supporting Information

**Fig. S1** Immunophenotype and *in vitro* differentiation of mesenchymal stromal cells.

**Fig. S2** Long-term culture of mesenchymal stromal cells.

**Fig. S3** β-galactosidase staining and expression of p16 in early and late mesenchymal stromal cell passages.

**Fig. S4** Methylation changes in independent donor samples and during long-term culture.

**Table S1** Differentially methylated CpGs upon long-term culture.

**Table S2** Gene ontology analysis of methylation changes in long-term culture.

**Table S3** Differentially methylated CpGs upon aging.

**Table S4** Gene ontology analysis of methylation changes upon aging.

**Table S5** Primer sequences.

As a service to our authors and readers, this journal provides supporting information supplied by the authors. Such materials are peer-reviewed and may be re-organized for online delivery, but are not copy-edited or typeset. Technical support issues arising from supporting information (other than missing files) should be addressed to the authors.
