# Supplementary figures and images for: DNA methylation pattern changes upon long-term culture and aging of human mesenchymal stromal cells
Source: Aging Cell. 2010 Feb;9(1):54–63. doi: 10.1111/j.1474-9726.2009.00535.x (PMC2814091; doi:10.1111/j.1474-9726.2009.00535.x)

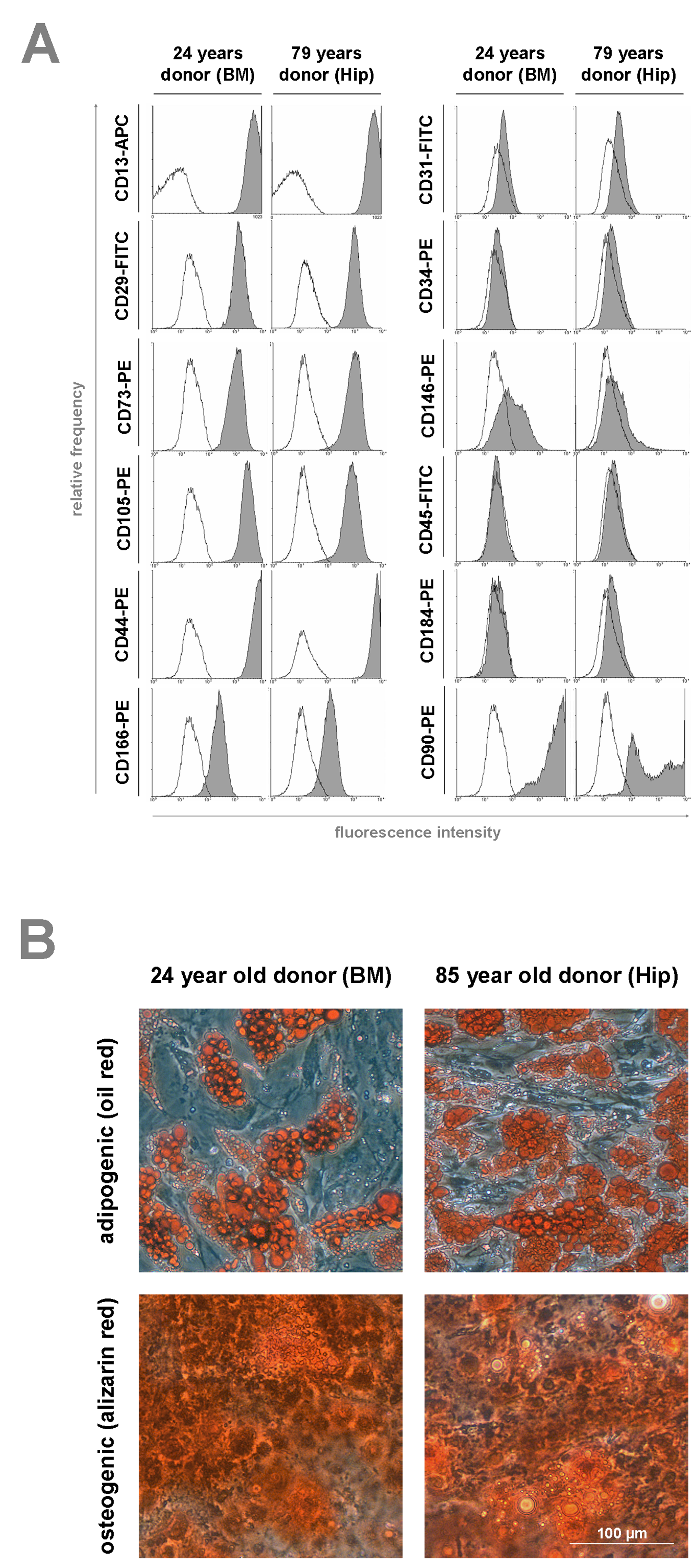

Supplement: Supplementary file 1 [file ace0009-0054-SD1.tif]

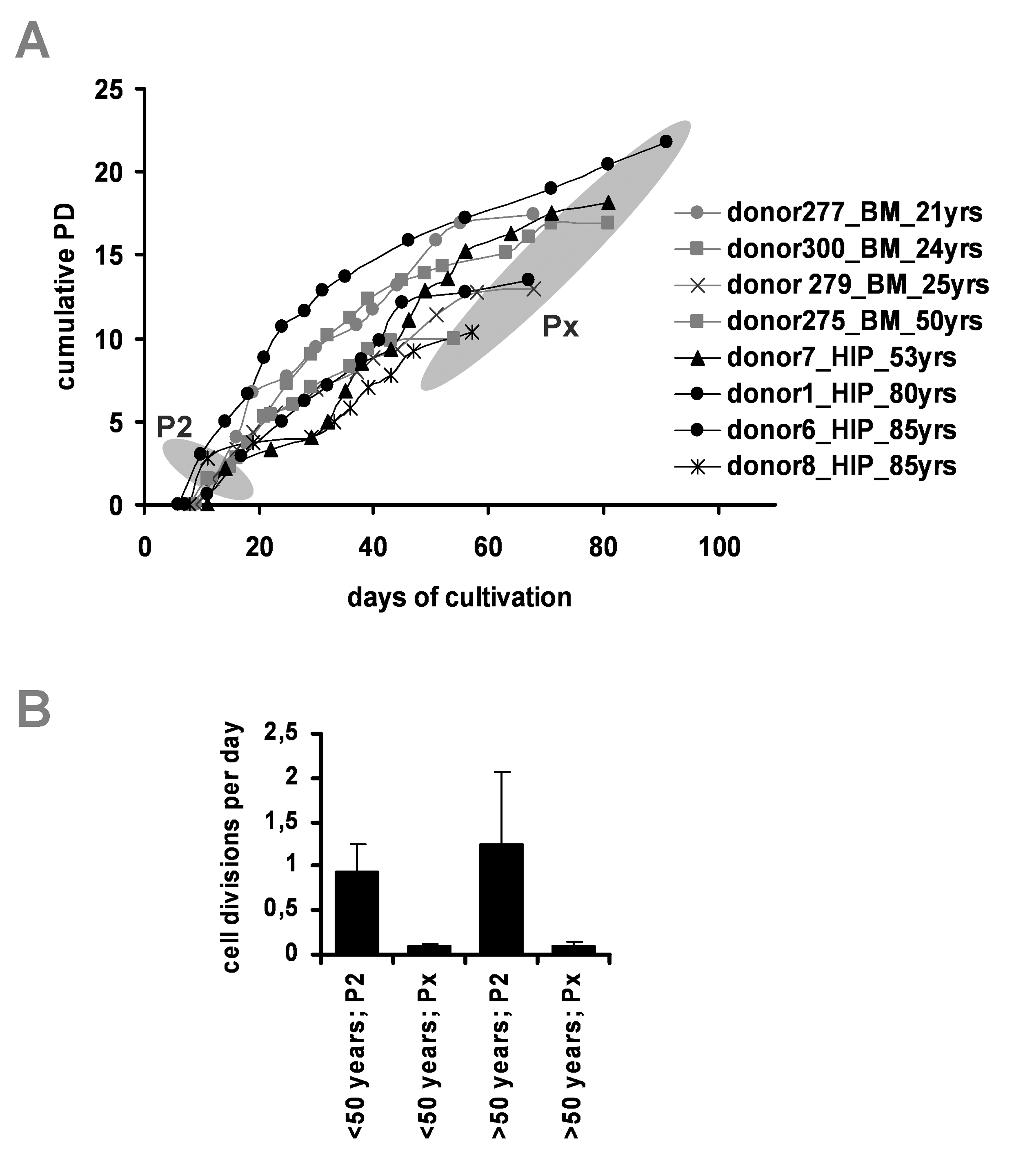

Supplement: Supplementary file 2 [file ace0009-0054-SD2.tif]

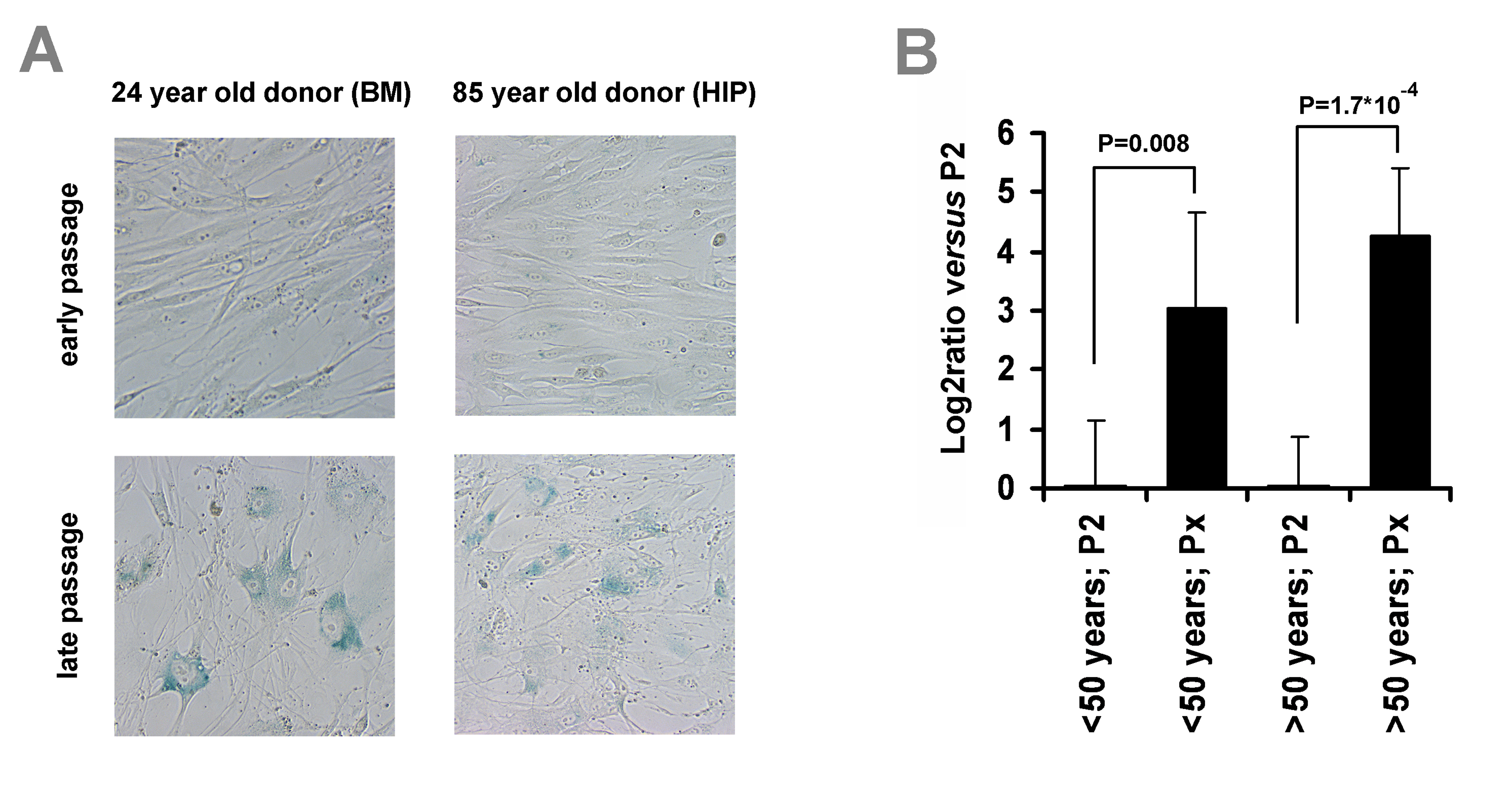

Supplement: Supplementary file 3 [file ace0009-0054-SD3.tif]

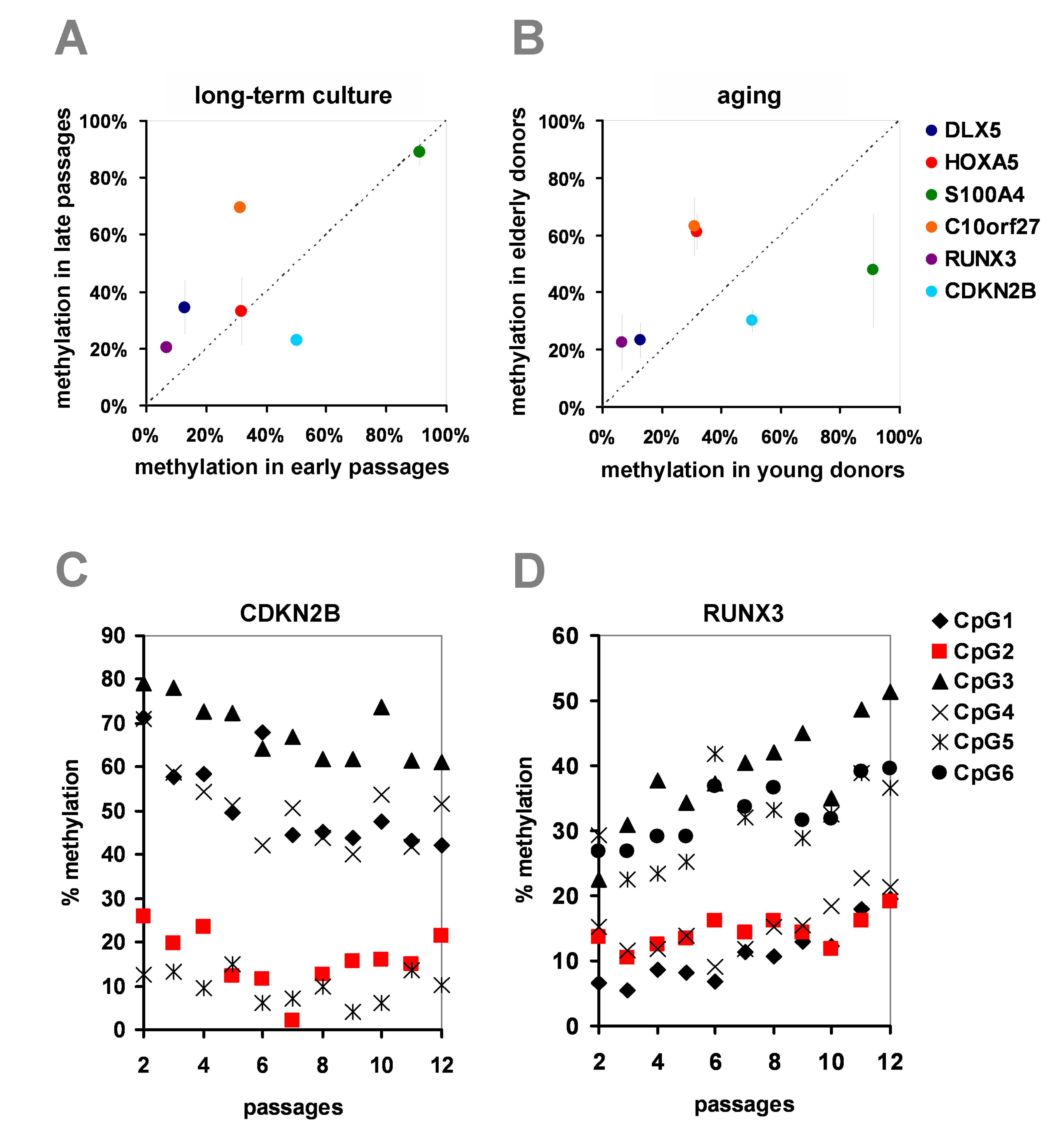

Supplement: Supplementary file 4 [file ace0009-0054-SD4.tif]
