# Supplementary material for: DNA methylation pattern changes upon long-term culture and aging of human mesenchymal stromal cells
Source: Aging Cell. 2010 Feb;9(1):54–63. doi: 10.1111/j.1474-9726.2009.00535.x (PMC2814091; doi:10.1111/j.1474-9726.2009.00535.x)
Supplement: Supplementary file 9 [file ace0009-0054-SD9.doc]

**Table S5: Primer sequences**

| **Gen** | **Amplicon length (bp)** | **Forward Primer** | **Reverse Primer** |
| --- | --- | --- | --- |
| *Primers for PCR amplification for pyrosequencing* | | | |
| CDKN2B | 308 | tgttagggagggttttttggata | Biotin-tccactcctccttcctataaatctca |
| S100A4 | 138 | gggtgtttatttgggaataggag | Biotin-ccaaccaaaccctacccataaca |
| DLX5 | 174 | Biotin-aattagaagtgtagtttaggtaggtttagtgtat | cccaaaactatttattccaactttca |
| HOXA5 | 223 | Biotin-ggtgtttaagtaggagggaattaagtatatgttt | ccctcaccaaactataatctccataat |
| RUNX3 | 144 | tagggttttgggtggggagtt | Biotin-aaaaaccaccaacccctcttc |
| C10orf27 | 243 | Biotin-gagttgtgggaatagggtaatttagta | tccacaaaacttccctttctacctc |
|  |  |  |  |
| *Primers for pyrosequencing reaction* | | | |
| CDKN2B |  | gagaagttgtagggtatttg |  |
| S100A4 |  | aataggaggtttggtttta |  |
| DLX5 |  | cccaaaactatttattcca |  |
| HOXA5 |  | ccataattatacaactaataatcc |  |
| RUNX3 |  | gtggggagttgtggttag |  |
| C10orf27 |  | tccaaaatccatctcctc |  |
|  |  |  |  |
| *Primers for qRT-PCR* | | | |
| GAPDH | 142 | TTCGTCATGGGTGTGAACCA | CTGTGGTCATGAGTCCTTCCA |
| CDKN2B | 274 | CCCAACTCCACCAGATAGCA | GGGATTTCCGCATCCTAGCA |
| S100A4 | 273 | CCTGGATGTGATGGTGTCCA | CTGGGCTGCTTATCTGGGAA |
| DLX5 | 233 | TGGTGGTAGGAGCTAGCGTA | GCTATGCACCATCCGTCTCA |
| HOXA5 | 216 | TATAGACGCACAAACGACCG | AGATCCATGCCATTGTAGCC |
| RUNX3 | 320 | GGTTTCTCTTGGCCTGTCCT | GCTGCCGTCACTTTTTGTCA |
| C10orf27 | 268 | TTCCACCACCAGTTCGTGAG | CACAATCCCTGGGATCACCA |
| p16 | 268 | CAACGCACCGAATAGTTACG | AGCACCACCAGCGTGTC |
